# Reporting quality of randomized controlled trials examining nutritional interventions in mild cognitive impairment

**DOI:** 10.3389/fnut.2026.1785846

**Published:** 2026-03-17

**Authors:** Lin Xu, Kun Tan, Bangmin Zhou, Xintong Tang, Jiajie Yu

**Affiliations:** 1 The First People’s Hospital of Shuangliu District, West China Airport Hospital of Sichuan University, Chengdu, China; 2 Sichuan Provincial Health Information Center, Chengdu, China; 3 The First Affiliated Hospital of Traditional Chinese Medicine of Chengdu Medical College, Xindu Hospital of Traditional Chinese Medicine, Chengdu, China; 4 Clinical Epidemiology and Evidence-Based Medicine Center, West China Hospital, Sichuan University, Chengdu, China

**Keywords:** CONSORT, mild cognitive impairment, nutrition, randomized controlled trial, reporting quality

## Abstract

**Background:**

Nutritional strategies are increasingly recognized as critical interventions for preventing or delaying the progression of Mild Cognitive Impairment (MCI) to dementia. While the randomized controlled trial (RCT) is considered the gold standard for evaluating healthcare interventions, nutrition-related trials in MCI populations are susceptible to specific methodological biases. Adequate reporting of these methodological domains is critical for assessing internal validity and reproducibility.

**Methods:**

We conducted a cross-sectional survey of English-language RCTs and systematically searched PubMed, EMBASE, and Web of Science from inception to March 15, 2025, with an updated search in August 2025. Reporting quality was evaluated using a modified composite checklist comprising 37 items from the CONSORT 2010 Statement and selected candidate items proposed for the nutritional extension (draft recommendations). We calculated an overall adherence score and performed multivariable linear regression to identify study characteristics associated with reporting quality.

**Results:**

A total of 75 trials were included. The mean overall adherence score was 20.4 (SD 5.2). While reporting of eligibility criteria (*n* = 71, 94.7%) and baseline characteristics (*n* = 72, 96.0%) was adequate, critical methodological domains were frequently under-reported. Only 25 trials (33.3%) described allocation concealment, 34 (45.3%) detailed blinding procedures, and 8 (10.7%) provided sufficient details on trial implementation. Notably, despite the target population, less than half of the trials (*n* = 32, 42.7%) reported data on intervention adherence or acceptability, and only 10 trials (13.3%) explicitly distinguished between statistical significance and clinical relevance. Multivariable analysis indicated that publication year (>2020), trial registration, and protocol availability were independently associated with higher reporting scores.

**Conclusion:**

Reporting quality in nutritional trials for MCI remains suboptimal, particularly in domains essential for interpreting trial validity, such as randomization, blinding, and adherence verification. The frequent omission of these details limits the ability to distinguish intervention effects from placebo responses or poor compliance. Rigorous adherence to comprehensive reporting standards, including the forthcoming CONSORT extension for nutrition, is necessary to improve the reliability and reproducibility of evidence in this field.

## 1 Introduction

Alzheimer’s disease (AD) is the leading cause of dementia, resulting in a significant loss of autonomy and independence in the elderly (1). Currently, pharmacological interventions for AD remain limited (2). Mild cognitive impairment (MCI), characterized by a decline in memory, attention, and cognitive function beyond that expected for age and education, represents a critical prodromal phase (3–5). Epidemiological studies estimate the prevalence of MCI in individuals older than 65 years to range from 3% to 19%. While some individuals with MCI may remain stable or revert to normal cognition, over 50% progress to dementia within 5 years (6). Consequently, early identification and management of MCI are essential for alleviating symptoms and delaying disease progression.

Given the limited efficacy of pharmacotherapy, identifying modifiable risk factors to prevent cognitive decline has attracted increasing attention (7). In recent years, the role of nutrition in preserving cognitive function has become a major area of investigative interest (8, 9). While the randomized controlled trial (RCT) is recognized as the gold standard for evaluating healthcare interventions, nutritional interventions differ fundamentally from pharmacological models (10, 11). In addition to the complexity of the food matrix and nutrient interactions, trials in MCI populations face specific methodological challenges: the difficulty of implementing effective blinding to minimize placebo effects in subjective cognitive outcomes, and the challenge of verifying rigorous adherence in older adults with memory deficits. Additionally, cognitive responses to nutritional changes are often subtle and require long durations to manifest (12–14). Consequently, without transparent reporting of intervention details, background diet, and compliance verification, it is difficult to determine whether a null result stems from biological inefficacy or suboptimal trial execution. Thus, adherence to rigorous, field-specific reporting standards is a prerequisite for evidence translation.

To improve the transparency and quality of trial reporting, the Consolidated Standards of Reporting Trials (CONSORT) group published the CONSORT Statement in 1996 (15), with subsequent updates in 2001 (16), 2010 (17), and 2025 (18). However, as CONSORT was originally developed for pharmacological interventions, its applicability to nutritional trials is somewhat limited. To address this gap, the Federation of European Nutrition Societies (FENS) called for a nutrition-specific extension (19). This initiative led to the development of the CONSORT Extension for Nutrition (CONSORT-Nut), with recommendations drafted to complement the standard checklist (20).

Despite the availability of these guidelines, the quality of reporting in nutritional trials for cognitive impairment remains unassessed. Therefore, the present study was designed as a cross-sectional survey to investigate the reporting quality of RCTs assessing nutritional interventions in MCI populations. We examined the extent to which these trials adhered to a modified composite checklist developed for this study, integrating items from the CONSORT 2010 Statement with selected nutrition-specific candidate items drawn from the draft CONSORT-Nut recommendations.

## 2 Methods

### 2.1 Study selection

Studies were considered eligible if they were randomized controlled trials (RCTs) conducted in participants with mild cognitive impairment (MCI), published as full-text articles in English, and represented the primary analysis of the trial. Detailed inclusion and exclusion criteria are provided in Box.

**Table:** 

	Inclusion criteria	Exclusion criteria
**Population**	Participants diagnosed with MCI using established criteria (e.g., Petersen criteria, NIA-AA, DSM-5)	Studies primarily include cognitively normal older adults, or dementia patients.
**Intervention**	Diet- and nutrition-related interventions, including: (1) whole foods/food products; (2) complete diets or dietary patterns; (3) enteral/parenteral nutrition formulas; (4) nutrient supplementation (single/multiple nutrients, bioactive compounds, plant-derived substances); (5) Multicomponent interventions (e.g., nutrition, nutritional education or counseling, combined with exercise or pharmacological treatments) also eligible.	Interventions limited to pharmaceuticals or herbal medicines.
**Comparator**	Any control condition (e.g., placebo, usual care, alternative intervention).	
**Outcomes**	Any clinical, cognitive, or nutritional outcomes	
**Study design**	Randomized controlled trials (self-identified as RCTs), including parallel RCT, crossover RCT and Factorial RCT	Secondary or *post hoc* analyses of RCTs; pilot/feasibility trials; duplicate reports from same trial (only main trial report included).

Box Inclusion and exclusion criteria.

### 2.2 Data sources and study selection

We systematically searched PubMed, EMBASE, and the Web of Science Core Collection from inception to March 15, 2025, with an update performed in August 2025. The search strategy included terms and their variants related to mild cognitive impairment, nutrition/diet, and randomized controlled trials, with no restrictions on publication date. The full search strategy is detailed in Supplementary Appendix 1. Additionally, reference lists of relevant systematic reviews on nutritional interventions for MCI were manually screened to identify potentially eligible trials.

All retrieved studies were imported into EndNote X9 and duplicates were removed before screening. Two reviewers independently screened titles/abstracts and full texts. Discrepancies were resolved through discussion or adjudication by a third reviewer.

### 2.3 Data extraction

Two groups of reviewers independently extracted data from the eligible studies using pilot-tested forms, with discrepancies resolved through discussion. The following study characteristics were collected: publication year, number of authors, sample size, journal category (nutrition, general medicine), country, study duration, multi-centers, CONSORT endorsement, protocol, data sharing, funding source (non-industry, industry, none, or not specified), and conflict of interest (yes, no, or not specified). CONSORT endorsement was identified by reviewing the journals’ Instructions for Authors.

We also extracted PICO-related information, including diagnostic criteria for MCI (e.g., Petersen criteria, DSM-5, NIA-AA), interventions details (e.g., dietary patterns, specific foods, supplements), comparators (e.g., placebo, usual care, alternative intervention), and outcomes (e.g., cognitive function, biochemical markers, neuroimaging).

We evaluated reporting quality using a modified composite checklist developed for this survey (Supplementary Appendix 2), anchored on the CONSORT 2010 Statement (17). To better reflect nutrition-specific considerations, we supplemented the standard items with selected candidate items proposed in the draft CONSORT Extension for Nutrition recommendations (20). We did not apply the full draft set; instead, we selected candidate items *a priori* based on their relevance to internal validity and interpretability in MCI nutrition trials. We prioritized items addressing intervention/comparator characterization, adherence/exposure and acceptability, and reporting that supports biological plausibility/mechanistic interpretation. Items were excluded if they were less directly related to internal validity, overlapped with CONSORT 2010, or required context- dependent judgments that could not be applied consistently across trials. This process resulted in a 37-item checklist tailored to the study aims. Supplementary Appendix 2 provides the full checklist and indicates the source of each item (CONSORT 2010 vs. draft CONSORT-Nut). Each trial was assessed for adherence to 37 reporting items and classified as “yes,” “partial yes,” “no,” or “not applicable.” Overall adherence was defined as the proportion of items answered “yes.” For scoring, one point assigned for “yes,” 0.5 point for “partial yes” and 0 point for “no”; items classified as “not applicable” were excluded from the scoring for that item. The total adherence scores therefore ranged from 0 to 37. Cohen’s kappa was calculated based on the two reviewers’ agreement on the adequacy of reporting for each CONSORT item. Interobserver agreement for the item-level CONSORT judgments was good (overall kappa = 0.77).

### 2.4 Data analysis

We calculated the proportion for categorical variables and mean (standard deviation) or median (interquartile range) for continuous variables. Univariable comparisons of reporting quality scores were conducted across nine prespecified study characteristics based on prior meta-research: sample size (dichotomized by the median of our sample), study duration, year of publication, journal type, trial registration, availability of protocol, CONSORT endorsement, funding source, and conflict of interest (COI) disclosure (21). Continuous variables with a non-normal distribution were analyzed using the Wilcoxon rank-sum test, whereas normally distributed variables were compared using the *t*-test. Multivariable linear regression was performed to evaluate associations between CONSORT scores and study characteristics; covariates with evidence of association in univariable analyses (*p* < 0.05) were included in the multivariable model. The distribution of CONSORT scores was assessed to confirm the assumption of normality.

## 3 Results

**FIGURE 1:**
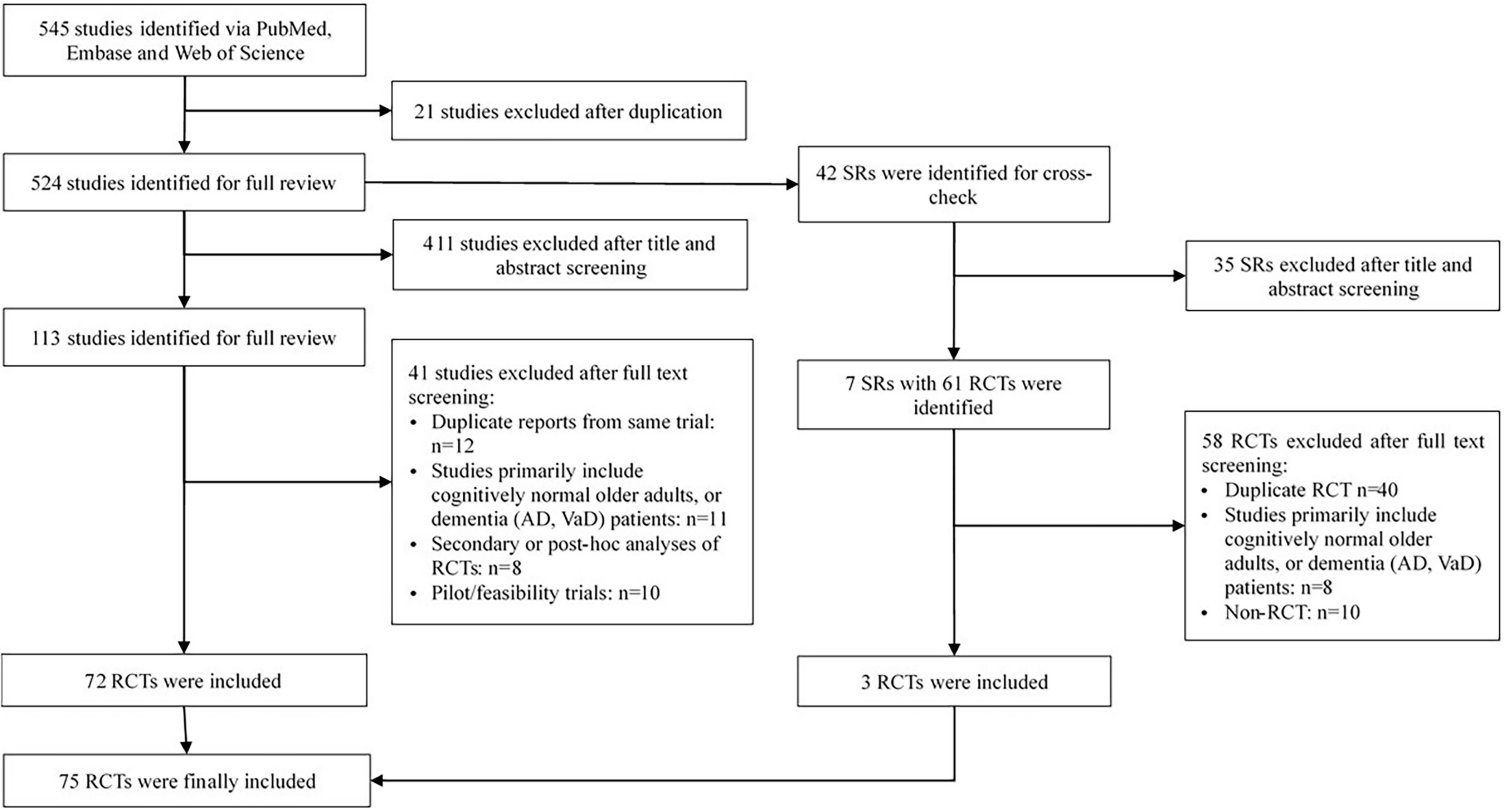
Study selection.

The database searches yielded 545 records. After screening titles and abstracts, 113 full-text articles were assessed for eligibility and 3 trials were identified reference lists of relevant systematic reviews. Finally, 75 trials met the inclusion criteria and were included in the analysis (Figure 1, Supplementary Appendix 3).

### 3.1 General information of included trials

Of those 75 trials, the median sample size was 78 (IQR: 42–152), and the median study duration was 6 months (IQR: 3–12). Twenty-seven trials (36.0%) were multicenter studies, and an equal number were published in nutrition-focused journals. While 46 trials (61.3%) mentioned CONSORT in the text, only 7 (9.3%) referenced a specific study protocol. Seventeen trials (22.7%) reported industry funding; 23 (30.7%) disclosed potential conflicts of interest.

Regarding PICO characteristics, the Petersen criteria were the most frequently used diagnostic tool for MCI (*n* = 30, 40.0%), followed by cognitive test cut-offs (*n* = 24, 32.0%) and DSM criteria (*n* = 8, 10.6%). Most trials evaluated dietary supplements (*n* = 50, 66.7%) and utilized a placebo control (*n* = 49, 65.3%). Sixty trials (80.0%) listed cognitive function as the primary outcome (Table 1).

**TABLE 1:** General characteristics of included 75 trials.

Characteristics	*n* (%)	PICO characteristics	*n* (%)
Number of authors*	9 (6, 11)	**Diagnosis of MCI**	
Sample sizes*	78 (42, 152)	Peterson criteria/modified Peterson criteria	30 (40.0)
**Publish year**		DSM-5/DSM-4	8 (10.6)
2005–2019	37 (49.3)	NIA-AA	5 (6.7)
2020–2025	38 (50.7)	IWG-MCI	3 (4.0)
**Type of journal**		Cognitive tests	24 (32.0)
Nutrition	27 (36.0)	Others	5 (6.7)
General	48 (64.0)	**Intervention**	
**Country**		Supplementation or supplements	50 (66.7)
China	19 (25.3)	Food	11 (14.6)
Japan	13 (17.3)	Nutrition education	6 (8.0)
Korea	8 (10.7)	Multicomponent interventions	5 (6.7)
USA	7 (9.3)	Dietary patterns	2 (2.7)
Others	28 (37.4)	Nutrition formulas	1 (1.3)
Multi-center study	27 (36.0)	**Control**	
CONSORT endorsement	46 (61.3)	Placebo	49 (65.3)
Study duration (month)*	6 (3–12)	Different intervention	20 (26.7)
**Study design**		Usual care	3 (4.0)
Parallel RCT	71 (94.6)	No intervention	3 (4.0)
Crossover RCT	2 (2.7)	**Outcomes**	
Factorial RCT	2 (2.7)	Cognitive function	60 (79.9)
**Funding sources**		Biochemical measurements	5 (6.7)
Industry	17 (22.7)	Neuroimaging	5 (6.7)
Non-industry	43 (57.3)	Severity of disease	2 (2.7)
No funding	5 (6.7)	Quality of life	1 (1.3)
Not specified	10 (13.3)	Others	2 (2.7)
**Conflict of interest**			
Yes	23 (22.7)		
No	42 (56.0)		
Not specified	10 (13.3)		
**Pre-registered**	47 (62.7)		
**Protocol**	7 (9.3)		
**Data sharing**	24 (32.0)		

The symbol “*” is expressed as the median and interquartile range (IQR).

### 3.2 Reporting quality of included trials

**FIGURE 2:**
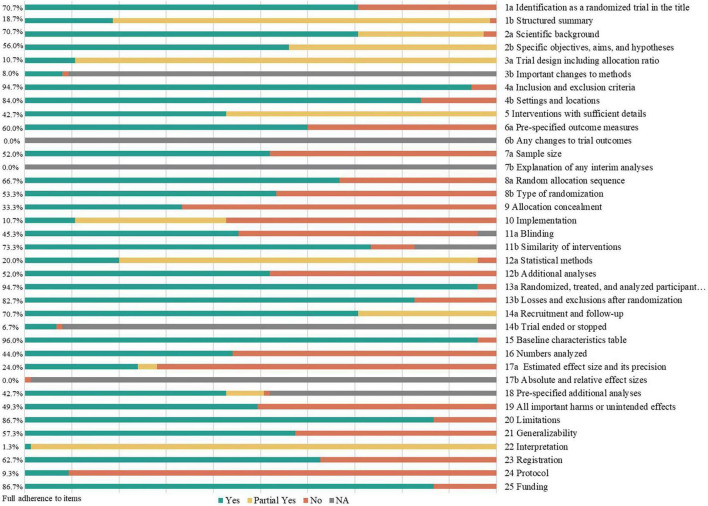
Adherence of included trials (the full wording, definitions, and scoring criteria for all 37 checklist items are provided in Supplementary Appendix 2).

Adherence to the reporting items varied substantially across domains (Figure 2). Among the 37 items, only seven were adequately reported by more than 80% of trials: eligibility criteria (94.7%), setting and location (84.0%), participant flow (94.7%), baseline demographics (96.0%), losses and exclusions after randomization (82.7%) limitations (86.7%), and funding sources (86.7%).

Conversely, reporting in several key methodological domains was suboptimal. Only eight trials (10.7%) provided a complete description of the trial design (e.g., allocation ratio) or implementation details (e.g., personnel generating the allocation sequence). Statistical methods regarding analysis sets were specified in 15 trials (20.0%), and effect estimates with precision (e.g., confidence intervals) were reported in 18 (24.0%). Intervention details regarding acceptability, adherence, and tolerance were provided in 32 trials (42.7%).

Regarding randomization, while 50 trials (66.7%) reported the sequence generation method, only 25 (33.3%) described allocation concealment, and 34 (45.3%) detailed blinding procedures. Furthermore, important post-randomization changes were rarely reported: only 6 (8.0%) reported methodological changes, and 5 (6.7%) explained reasons for early termination. No trials reported changes to outcomes, interim analyses, or absolute effect sizes for binary outcomes. Within the discussion sections, reporting on interpretation remained limited. Only 11 trials (14.7%) discussed relevant aspects of the active constituent of the intervention, and 10 (13.3%) explicitly distinguished between statistical significance and clinical relevance.

The mean overall score for the modified CONSORT checklist (37 items) was 20.4 (SD = 5.2). Trials published after 2020 scored significantly higher than those published earlier (22.4 ± 4.7 vs. 18.3 ± 5.0; *P* < 0.001). Univariable analysis indicated that larger sample size (>78), trial registration, protocol availability, CONSORT endorsement, funding disclosure, and COI statements were associated with higher scores (Table 2). In the multivariable linear regression model, publication year (>2020), trial registration, and protocol availability remained independent predictors of better reporting quality (Table 3).

**TABLE 2:** Reporting score of 75 RCTs in MCI.

Characteristics	Categories (*n*)	Mean (SD)	*P*
Sample size	<median (78 patients) (36)	18.8 (4.8)	0.013
	≥median (39)	21.8 (5.2)	
Study duration	<median (6 months) (32)	19.5 (5.1)	0.235
	≥median (43)	21.0 (5.3)	
Publish year	2005–2019 (37)	18.3 (5.0)	<0.001
	2020–2025 (38)	22.4 (4.7)	
Type of journal	Nutrition (27)	19.5 (5.1)	0.317
	General (48)	20.8 (5.3)	
Registration	Yes (47)	22.4 (4.6)	<0.001
	No (28)	17.0 (4.5)	
Protocol	Yes (6)	28.9 (2.4)	<0.001
	No (69)	19.6 (4.7)	
CONSORT endorsement	Yes (46)	21.4 (4.8)	0.035
	No (29)	18.8 (5.5)	
Funding	Reported (65)	21.1 (5.0)	0.002
	Not specified (10)	15.7 (4.5)	
Conflict of interest	Reported (65)	21.0 (4.8)	0.009
	Not specified (10)	16.4 (6.1)	

**TABLE 3:** Multivariable linear regression analysis of factors associated with adherence of reporting.

Factors	β coefficient (95% CI)	*P*-value
Publish year (2005–2019 versus 2020–2025)	2.00 (0.02, 3.99)	0.048
Sample size (<78 versus ≥78)	1.45 (−0.42, 3.32)	0.127
CONSORT endorsement (No versus Yes)	1.06 (−0.90, 3.02)	0.283
Registration (No versus Yes)	2.56 (0.36, 4.77)	0.023
Funding source (No versus Yes)	1.32 (−1.72, 4.37)	0.389
COI disclosure (No versus Yes)	1.93 (−1.08, 4.94)	0.205
Protocol reported (No versus Yes)	6.93 (3.42, 10.51)	<0.001

## 4 Discussion

This study provides a systematic evaluation of reporting quality in randomized controlled trials examining nutritional interventions for mild cognitive impairment. Reporting adherence improved among trials published after 2020, however, overall quality remained suboptimal, with a mean adherence score of 20.4 out of 37. Key methodological elements, including randomization procedures, allocation concealment, blinding, and protocol availability, were frequently insufficiently reported. Incomplete reporting in these domains constrains assessment of internal validity and limits the ability to distinguish intervention effects from potential sources of bias. Although our reporting assessment applied CONSORT 2010, the core methodological reporting items most relevant to internal validity (e.g., allocation concealment and blinding) remain conceptually consistent in the CONSORT 2025 update; therefore, our overall interpretation of the key reporting gaps is unlikely to be materially altered. Nevertheless, CONSORT 2025 introduces additional emphasis on open-science practices (e.g., data sharing) and patient/public involvement, which were not fully captured in our assessment and might have highlighted further reporting deficiencies.

A primary concern identified in this survey is the poor reporting of blinding (45.3%) and allocation concealment (33.3%). In trials of nutritional interventions for mild cognitive impairment, outcomes commonly rely on subjective cognitive assessments, such as Petersen criteria or composite cognitive test batteries. In this context, limited blinding increases susceptibility to performance and detection bias (21). Individuals with mild cognitive impairment often retain awareness of treatment allocation, and expectations regarding intervention benefit may influence cognitive performance when participants know they are receiving an active dietary intervention. Although blinding is difficult to implement in whole-food interventions because of unavoidable sensory differences between intervention and control conditions, this does not remove the requirement to clearly report whether blinding was attempted and how potential bias was addressed. Similarly, poor reporting of allocation concealment limits assessment of internal validity, as it remains unclear whether group assignment was protected against selection bias, including preferential enrollment of participants with more favorable prognostic characteristics. For clinicians and guideline panels, unclear blinding and concealment make it difficult to judge whether observed cognitive benefits (or harms) reflect true intervention effects or bias, thereby weakening the basis for clinical recommendations.

Only 10.7% of trials adequately reported details related to trial implementation. In nutritional interventions involving older adults with cognitive impairment, participant adherence is a central determinant of intervention effectiveness (22, 23). Cognitive limitations associated with mild cognitive impairment may interfere with sustained adherence to prescribed dietary regimens, and limited reporting of adherence assessment and support reduces the interpretability of trial findings (24). Consequently, it is often unclear whether null or modest effects reflect limited intervention efficacy or insufficient exposure due to suboptimal adherence. Poor reporting of implementation characteristics (e.g., adherence targets, fidelity monitoring, dietary counseling intensity, and co-interventions) also limits reproducibility and real-world applicability, making it difficult for clinicians and guideline panels to infer the intensity, key components, and delivery conditions required to translate these interventions into practice. In addition, only 11 trials (14.7%) reported relevant aspects of the active constituent(s), which constrains mechanistic interpretation and complicates comparisons across studies, because observed effects may arise from specific nutrients/bioactives, the food matrix, or broader dietary pattern changes.

Only 9.3% of trials cited a prespecified study protocol. The absence of protocol information restricts evaluation of outcome selection and analytical decisions and complicates assessment of selective reporting (25). While trial registration was associated with higher reporting scores in multivariable analyses, registration records alone often lack sufficient detail to fully document prespecified analytical plans. Accordingly, future trials should supplement registration with a publicly accessible, time-stamped statistical analysis plan (SAP) detailing prespecified outcomes and analyses, thereby facilitating assessment of selective reporting. The association between more recent publication year (>2020) and improved reporting quality is consistent with increased editorial attention to reporting standards, although absolute adherence levels remain modest.

Greater emphasis on reporting considerations during the trial design phase may improve the interpretability of future studies (26). Integration of nutrition-specific reporting items (such as those proposed in the draft extension) during protocol development, rather than solely at manuscript submission, may facilitate more complete documentation of key methodological features (27, 28). Protocols should specify approaches for monitoring adherence and accounting for background diet, given their importance in dietary interventions involving cognitively impaired populations (21). Clear reporting of randomization procedures, blinding status (or explicit acknowledgment of open-label designs), and effect estimates with appropriate measures of precision would further strengthen the evidence base.

These findings are consistent with previous evaluations of reporting quality in nutritional research, which have reported similar gaps in key methodological domains, particularly allocation procedures, protocol availability, and outcome specification (9, 21, 29). Improvements over time parallel trends observed in other areas of clinical research, likely reflecting broader dissemination of CONSORT guidance. Nevertheless, reporting quality in nutritional trials remains lower than that typically observed in pharmacological randomized controlled trials. Trials in mild cognitive impairment are often smaller, shorter in duration, and more heterogeneous in intervention design, factors that may contribute to variability in reporting practices.

This study has several strengths. Application of the CONSORT 2010 Statement together with selected candidate items from the draft nutrition extension enabled a structured assessment tailored to nutritional intervention trials. Focusing on randomized controlled trials in mild cognitive impairment also allowed examination of reporting issues specific to this clinical context. Several limitations should be considered. First, this study addressed reporting quality rather than the quality of trial conduct, and well-conducted studies may not always be adequately reported. Second, our assessment was based on the CONSORT 2010 Statement, as pilot data extraction was completed before publication of the 2025 update, although core methodological requirements remain consistent across versions. Third, we used a modified composite checklist incorporating selected candidate items from the draft nutrition extension. As the full draft recommendations were not applied, our findings reflect key reporting domains relevant to MCI trials (e.g., adherence, blinding) rather than compliance with the forthcoming official CONSORT-Nut guidelines. Fourth, restriction to English-language publications may have led to omission of relevant studies published in other languages.

## 5 Conclusion

In conclusion, despite modest improvements over time, the reporting quality of randomized controlled trials evaluating nutritional interventions for mild cognitive impairment remains suboptimal, particularly in blinding, allocation concealment, and protocol transparency. Given the reliance on subjective cognitive outcomes and the challenges associated with dietary adherence in this population, complete and transparent reporting is necessary to support reliable evidence synthesis. More consistent application of comprehensive and nutrition-specific reporting standards, including the forthcoming CONSORT extension, may strengthen the contribution of future trials to clinical guidance on dementia prevention.

## Data Availability

The raw data supporting the conclusions of this article will be made available by the authors, without undue reservation.
