# Supplementary material for: Reporting quality of randomized controlled trials examining nutritional interventions in mild cognitive impairment
Source: Front Nutr. 2026 Mar 17;13:1785846. doi: 10.3389/fnut.2026.1785846 (PMC13035510; doi:10.3389/fnut.2026.1785846)
Supplement: Supplementary file 1 [file Table_1.docx]

Appendix 1 Search strategy

**PubMed**

#1 "Cognitive Dysfunction"[MeSH Terms] OR "cognitive dysfunction*"[Title/Abstract] OR "mild cognitive impairment*"[Title/Abstract] OR "cognitive defect"[Title/Abstract] OR "cognitive declin*"[Title/Abstract] OR "cognitive impairment*"[Title/Abstract] OR "mild neurocognitive disorder*"[Title/Abstract] OR "mental deterioration*"[Title/Abstract] OR "age associated cognitive decline"[Title/Abstract] OR "pre-dementia"[Title/Abstract] OR MCI[Title/Abstract]

#2 "nutritional sciences"[MeSH Terms] OR "nutritional physiological phenomena"[MeSH Terms] OR "nutrition assessment"[MeSH Terms] OR "nutritional support"[MeSH Terms] OR "nutrition therapy"[MeSH Terms] OR "nutrition policy"[MeSH Terms] OR "nutritional and metabolic diseases"[MeSH Terms] OR "nutrition*"[Title/Abstract] OR "diet"[Title/Abstract] OR "breastfeed*"[Title/Abstract] OR "breast feed*"[Title/Abstract] OR "lactation"[Title/Abstract] OR "bottle feed*"[Title/Abstract] OR "complementary feeding"[Title/Abstract] OR "weaning"[Title/Abstract] OR "enteral"[Title/Abstract] OR "parenteral"[Title/Abstract] OR "overweight"[Title/Abstract] OR "obese"[Title/Abstract] OR "obesity"[Title/Abstract] OR "overnutrition"[Title/Abstract] OR "over nutrition"[Title/Abstract] OR "wasted"[Title/Abstract] OR "wasting"[Title/Abstract] OR "stunting"[Title/Abstract] OR "stunted"[Title/Abstract] OR "underweight"[Title/Abstract] OR "undernutrition"[Title/Abstract] OR "under nutrition"[Title/Abstract] OR "body weight"[Title/Abstract] OR "growth monitoring"[Title/Abstract] OR "Food"[Title/Abstract] OR "food labeling"[MeSH Terms] OR "food assistance"[MeSH Terms] OR "supplementary feeding"[Title/Abstract] OR "diet therapy"[MeSH Terms] OR "food and beverages"[MeSH Terms] OR "vegetable*"[Title/Abstract] OR "fruit*"[Title/Abstract] OR "meat"[Title/Abstract] OR "dairy"[Title/Abstract] OR "dietary fat*"[Title/Abstract] OR "starch*"[Title/Abstract] OR "cereal"[Title/Abstract] OR "food drug interactions"[MeSH Terms] OR "food supply"[MeSH Terms] OR "feeding behavio*"[Title/Abstract] OR "eating behavio*"[Title/Abstract] OR "food hypersensitivity"[MeSH Terms] OR "food deprivation"[MeSH Terms] OR "food, organic"[MeSH Terms] OR "micronutrient*"[Title/Abstract] OR "vitamin*"[Title/Abstract] OR "minerals"[Title/Abstract] OR "trace element*"[Title/Abstract] OR "trace metal*"[Title/Abstract] OR "macronutrient*"[Title/Abstract] OR "carbohydrate*"[Title/Abstract] OR "dietary protein*"[Title/Abstract] OR "saturated fat*"[Title/Abstract] OR "unsaturated fat*"[Title/Abstract] OR "mono unsaturated fat*"[Title/Abstract] OR "monounsaturated fat*"[Title/Abstract] OR "poly unsaturated fat*"[Title/Abstract] OR "polyunsaturated fat*"[Title/Abstract] OR "trans fat*"[Title/Abstract] OR "dietary fibre"[Title/Abstract] OR "dietary fiber"[Title/Abstract] OR "dietary salt"[Title/Abstract] OR "table salt"[Title/Abstract] OR "soft drink"[Title/Abstract] OR "fruit juice"[Title/Abstract] OR "vegetable juice"[Title/Abstract] OR "milk"[Title/Abstract] OR "tea"[Title/Abstract] OR "coffee"[Title/Abstract] OR "energy drink*"[Title/Abstract] OR "carbonated beverage*"[Title/Abstract] OR "carbonated drink*"[Title/Abstract] OR "prebiotics"[Title/Abstract] OR "probiotics"[Title/Abstract] OR "glycemic load"[Title/Abstract] OR "glycemic index"[Title/Abstract] OR "glycaemic load"[Title/Abstract] OR "glycaemic index"[Title/Abstract] OR "calories"[Title/Abstract] OR "kilocalories"[Title/Abstract] OR "kilojoules"[Title/Abstract]

#3 "randomized controlled trial"[Publication Type] OR "controlled clinical trial"[Publication Type] OR "randomized"[Title/Abstract] OR "placebo"[Title/Abstract] OR "clinical trials as topic"[MeSH Terms:noexp] OR "randomly"[Title/Abstract] OR "trial"[Title]

#4 "animals"[MeSH Terms] NOT "humans"[MeSH Terms]

#5 #1 AND #2 AND #3

#6 #5 NOT #4

**Embase**

#1 ‘Cognitive Dysfunction’/exp OR ‘cognitive dysfunction’:ab,ti OR ‘mild cognitive impairment’:ab,ti OR ‘cognitive defect’:ab,ti OR ‘cognitive decline’:ab,ti OR ‘cognitive impairment’:ab,ti OR ‘mild neurocognitive disorder’:ab,ti OR ‘mental deterioration’:ab,ti OR ‘age associated cognitive decline’:ab,ti OR ‘pre-dementia’:ab,ti OR MCI:ab,ti

#2 ‘Nutritional Science’/exp OR 'nutritional science':kw OR 'nutritional physiological phenomena':ab,ti OR ‘Nutrition’/exp OR 'nutritional assessment':kw OR 'nutritional support':kw OR 'diet therapy':kw OR ‘Diet Therapy’/exp OR (nutritional:kw AND 'metabolic disorder':kw) OR ‘Nutritional Disorder’/exp OR ‘Metabolic Disorder’/exp OR nutrition:ab,ti OR diet:ab,ti,kw OR feeding:ab,ti OR ‘Food’/exp OR 'dietary intake':ab,ti OR ‘Diet Restriction’/exp OR breastfeeding:ab,ti OR lactation:ab,ti OR 'bottle feeding':ab,ti OR 'complementary feeding':ab,ti OR weaning:ab,ti OR 'enteric feeding':ab,ti OR parenteral:ab,ti OR 'food intake':ab,ti OR 'nutritional status':ab,ti OR ‘Failure to Thrive’/de OR ‘Body Weight’/exp OR obesity:ab,ti OR 'obese patient':ab,ti OR overnutrition:ab,ti OR 'wasting syndrome':ab,ti OR stunting:ab,ti OR 'stunting syndrome':ab,ti OR malnutrition:ab,ti OR ‘malnutrition’/de OR 'body weight':ab,ti OR anthropometry:kw OR ('growth, development':ab,ti AND aging:ab,ti) OR food:ab,ti OR 'food packaging':kw OR ‘Food Packaging’/de OR 'food assistance':kw OR ‘Food Assistance’/de OR 'supplementary feeding':ab,ti OR ‘Food Insecurity’/exp OR vegetable:ab,ti OR fruit:ab,ti OR meat:ab,ti OR dairy:ab,ti OR 'fat intake':ab,ti OR starch:ab,ti OR cereal:ab,ti OR 'food drug interaction':ab,ti OR 'catering service':kw OR 'feeding behavior':ab,ti OR 'eating habit':ab,ti OR 'food allergy':kw OR 'food deprivation':kw OR ‘Food Deprivation’/exp OR 'organic food':ab,ti OR ‘Organic Food’/de OR 'micronutrient intake':ab,ti OR vitamin:ab,ti OR thiamine:ab,ti OR riboflavin:ab,ti OR 'nicotinic acid':ab,ti OR 'pantothenic acid':ab,ti OR pyridoxine:ab,ti OR pyridoxal:ab,ti OR pyridoxamine:ab,ti OR biotin:ab,ti OR 'folic acid':ab,ti OR cyanocobalamin:ab,ti OR choline:ab,ti OR retinol:ab,ti OR 'ascorbic acid':ab,ti OR tocopherol:ab,ti OR carotenoid:ab,ti OR carotene:ab,ti OR cryptoxanthin:ab,ti OR xanthophyll:ab,ti OR lycopene:ab,ti OR zeaxanthin:ab,ti OR mineral:ab,ti OR calcium:ab,ti OR chloride:ab,ti OR magnesium:ab,ti OR phosphorus:ab,ti OR potassium:ab,ti OR sodium:ab,ti OR sulfur:ab,ti OR 'trace element':ab,ti OR boron:ab,ti OR cobalt:ab,ti OR chromium:ab,ti OR copper:ab,ti OR fluoride:ab,ti OR iodine:ab,ti OR iron:ab,ti OR manganese:ab,ti OR molybdenum:ab,ti OR selenium:ab,ti OR zinc:ab,ti OR 'trace metal':ab,ti OR macronutrient:ab,ti OR carbohydrate:ab,ti OR 'protein intake':ab,ti OR 'saturated fatty acid':ab,ti OR 'unsaturated fatty acid':ab,ti OR 'monounsaturated fatty acid':ab,ti OR 'polyunsaturated fatty acid':ab,ti OR 'trans fatty acid':ab,ti OR 'dietary fiber':ab,ti OR 'salt intake':ab,ti OR 'soft drink':ab,ti OR 'fruit juice':ab,ti OR 'vegetable juice':ab,ti OR milk:ab,ti OR tea:ab,ti OR coffee:ab,ti OR 'energy drink':ab,ti OR 'carbonated beverage':ab,ti OR 'prebiotic agent':ab,ti OR 'probiotic agent':ab,ti OR 'glycemic index':ab,ti OR 'glycemic load':ab,ti OR calorie:ab,ti OR 'caloric intake':ab,ti

#3 'clinical trial'/de OR 'randomized controlled trial'/de OR 'controlled clinical trial'/de OR 'multicenter study'/de OR 'phase 3 clinical trial'/de OR ‘phase 4 clinical trial’/de OR randomization/exp OR 'single blind procedure'/de OR 'double blind procedure'/de OR 'crossover procedure'/de OR placebo/de OR 'randomi?ed controlled trial*$':ab,ti OR rct:ab,ti OR 'random* NEAR/2 allocat*':ab,ti OR 'single blind*':ab,ti OR 'double blind*':ab,ti OR ((treble:ab,ti OR triple:ab,ti) NEAR blind*:ab,ti) OR placebo*:ab,ti OR 'prospective study'/de

#4 #1 AND #2 AND #3

**Web of Science**

#1

#2 AK=("nutritional sciences") OR AK=("nutritional physiological phenomena" ) OR AK=("nutrition assessment") OR AK=("nutritional support") OR AK=(“nutrition”) OR AK=("nutrition therapy") OR AK=("nutritional and metabolic diseases") OR TS=("nutrition*") OR KP=(“nutrition”)) OR KP=(“diet”)) OR TS =("diet") OR TS =("feeding") OR TS =("dietary") OR TS =("breastfeed*") OR TS =("breast feed*") OR TS =("lactation") OR TS =("bottle feed*") OR TS =("complementary feeding") OR TS =("weaning") OR TS =("enteral") OR TS =("parenteral") OR AK=("feeding methods") OR TS =("nutritional status") OR KP=(“nutrition support”) OR TS =("overweight") OR TS =("obese") OR TS =("obesity") OR TS =("overnutrition") OR TS =("over nutrition") OR TS =("undernourished") OR TS =("overnourished") OR TS =("wasted") OR TS =("wasting") OR TS =("stunting") OR TS =( "stunted") OR TS =("underweight") OR TS =("undernutrition") OR TS =("under nutrition") OR TS =("body weight") OR TS =("anthropometry") OR AK=("body weights and measures") OR TS =("growth monitoring") OR TS =("food") OR AK=("food labeling") OR AK=("food assistance") OR TS =("supplementary feeding") OR AK=("diet therapy") OR AK=("food and beverages") OR TS =("vegetable*") OR TS =("fruit*") OR TS =("meat") OR TS =("dairy") OR TS =("dietary fat*") OR AB=("starch*") OR TS =( "cereal") OR AK=("food drug interactions") OR AK=("food supply") OR TS =("feeding behavio*") OR TS =("eating behavio*") OR TS =("food pattern*") OR TS =("food hypersensitivity") OR TS =("food deprivation") OR TS =("food, organic") OR TS =("micronutrient*") OR TS =("vitamin*") OR TS =("thiamin") OR TS =("riboflavin") OR TS =("niacin") OR TS =("pantothenic acid") OR TS =("pyridoxine") OR TS =("pyridoxal") OR TS =("pyridoxamine") OR TS =("biotin") OR TS =("folic acid") OR TS =("folate")) OR TS =("cyanocobalamin") OR TS =("choline") OR TS =("retinol") OR TS =("ascorbic acid") OR TS =("tocopherol") OR TS =("carotenoids") OR TS =("carotene") OR TS =("cryptoxanthin") OR TS =("lutein") OR TS =("lycopene") OR TS =("zeaxanthin") OR TS =("minerals") OR TS =("calcium") OR TS =("chloride") OR TS =("magnesium") OR TS =("phosphorus") OR TS =("potassium") OR TS =("sodium") OR TS =("sulphur") OR TS =("trace element*") OR TS =("boron") OR TS =("cobalt") OR TS =("chromium") OR TS =("copper") OR TS =("fluoride") OR TS =("iodine") OR TS =("iron") OR TS =("manganese") OR TS =("molybdenum") OR TS =("selenium") OR TS =("zinc") OR TS =("trace metal*") OR TS =("macronutrient*") OR TS =("carbohydrate*") OR TS =("dietary protein*") OR TS =("saturated fat*") OR TS =("unsaturated fat*" ) OR TS =("mono unsaturated fat*") OR TS =("monounsaturated fat*") OR TS =("poly unsaturated fat*") OR TS =("polyunsaturated fat*")OR TS =("trans fat*") OR TS =("dietary fibre") OR TS =("dietary fiber") OR TS =("dietary salt") OR TS =("table salt") OR TS =("soft drink") OR TS =("fruit juice") OR TS =("vegetable juice") OR TS =("milk") OR TS =("tea") OR TS =("coffee") OR TS =("energy drink*") OR TS =("carbonated beverage*") OR TS =("carbonated drink*") OR TS =("prebiotics") OR TS =("probiotics") OR TS =("glycaemic load") OR TS =("glycaemic index") OR TS =("calories") OR TS =("kilocalories") OR TS =("kilojoules") OR TS =("caloric intake") OR TS =("energy intake")

#3 TS =(randomised OR randomized OR randomisation OR randomisation OR placebo* OR (random*)

#4 #1 AND #2 AND #3

**Appendix 2 Composite reporting checklist used in the study (based on CONSORT 2010 and selected draft nutrition items)**

| Section/topic | No | CONSORT checklist item | Draft CONSORT-Nut candidate item^[20]^ | Reported |
| --- | --- | --- | --- | --- |
| **Title and abstract** | | | | |
|  | 1a | Identification as a randomized trial in the title | - Where possible, the type of dietary comparator should be described in the title | □ Yes  □ No |
|  | 1b | Structured summary of trial design, methods, results, and conclusions | - Include details of intervention and comparator - Clearly state the primary outcome - Specified the trial design (cluster, cross-over, parallel, etc) - Include treatment effects | □ Yes  □ Partial yes  □ No |
| **Introduction** | | | | |
| Background | 2a | Scientific background and explanation of rationale | - State the biological plausibility of the nutrition intervention and and/or behavioral, physiological, or molecular mechanism - Provide contextualization where relevant to current dietary recommendations or food intake in the population of interest | □ Yes  □ Partial yes  □ No |
| Objectives | 2b | Specific objectives, aims, and hypotheses   - Specific objectives, aims - Specific hypotheses |  | □ Yes  □ Partial yes  □ No |
| **Methods** | | | | |
| Trial design | 3a | Description of trial design (such as parallel, factorial) including allocation ratio | - The type of trial (e.g., parallel group, crossover, factorial, cluster) - The allocation ratio (N/A if a crossover design) - The study framework (superiority, equivalence, non-inferiority, exploratory) - The duration of washout period and consideration of carry over effect in cross trial - Duration of the trial should be appropriate for the primary outcomes - Potential confounders (baseline nutritional status and factors that could influence trial outcomes) | □ Yes  □ Partial yes  □ No |
|  | 3b | When applicable, important changes to methods after trial commencement (such as eligibility criteria), with reasons |  | □ Yes  □ No  □ Not applicable |
| Participants | 4a | The inclusion and exclusion criteria for participants/centers |  | □ Yes  □ No |
|  | 4b | Settings and locations where the data were collected (community, office practice, hospital clinic, or inpatient unit) |  | □ Yes  □ No |
| Interventions | 5 | The interventions for each group with sufficient details to allow replication | - Details of comparators - Details of the diet-related intervention - Acceptability, adherence and tolerance of intervention | □ Yes  □ Partial yes  □ No |
| Outcomes | 6a | Completely defined pre-specified primary and secondary outcome measures, including how and when they were assessed |  | □ Yes  □ No |
|  | 6b | When applicable, any changes to trial outcomes after the trial commenced, with reasons |  | □ Yes  □ No  □ Not applicable |
| Sample size | 7a | How sample size was determined (with alpha, ß, SD, ICC in cluster trial) |  | □ Yes  □ No |
|  | 7b | When applicable, explanation of any interim analyses and stopping guidelines |  | □ Yes  □ No  □ Not applicable |
| Randomization |  |  |  |  |
| - Sequence generation | 8a | Method used to generate the random allocation sequence |  | □ Yes  □ No |
|  | 8b | Type of randomization; details of any restriction (such as blocking and block size) |  | □ Yes  □ No |
| - Allocation concealment | 9 | Describing any steps taken to conceal the sequence (such as sequentially numbered containers) |  | □ Yes  □ No |
| - Implementation | 10 | Who generated the random allocation sequence, who enrolled participants, and who assigned participants to interventions |  | □ Yes  □ Partial yes  □ No |
| Blinding | 11a | Described who was blinded after assignment to interventions and how |  | □ Yes  □ No  □ N/A if it is not blinded |
|  | 11b | If relevant, a description of the similarity of interventions |  | □ Yes  □ No  □ Not applicable |
| Statistical methods | 12a | Statistical methods used to compare groups for primary and secondary outcomes | - An a priori statistical analysis plan - primary analysis should be based on ITT, with per-protocol analysis described in addition where relevant - Comparisons between intention-to-treat and per-protocol analyses - When applicable, adjust for stratification variables | □ Yes  □ Partial yes  □ No |
|  | 12b | Methods for additional analyses, such as subgroup analyses and adjusted analyses |  | □ Yes  □ No |
| **Results** | | | | |
| Participant flow | 13a | For each group, the numbers of participants who were randomly assigned, received intended treatment and were analyzed for the primary outcome |  | □ Yes  □ No |
|  | 13b | For each group, losses and exclusions after randomization, together with reasons |  | □ Yes  □ No |
| Recruitment | 14a | Dates defining the periods of recruitment and follow-up   - Periods of recruitment - Length of follow-up for outcomes |  | □ Yes  □ Partial yes  □ No |
|  | 14b | Why the trial ended or was stopped |  | □ Yes  □ No  □ Not applicable |
| Baseline data | 15 | A table showing baseline demographic and clinical characteristics for each group |  | □ Yes  □ No |
| Numbers analyzed | 16 | For each group, the number of participants included in each analysis and whether the analysis was by originally assigned groups |  | □ Yes  □ No |
| Outcomes and  estimation | 17a | For each primary and secondary outcome, results for each group, and the estimated effect size and its precision (such as 95% confidence interval)   - For primary outcome - For secondary outcomes |  | □ Yes  □ Partial yes  □ No |
|  | 17b | For binary outcomes, presentation of both absolute and relative effect sizes is recommended |  | □ Yes  □ No  □ N/A in none binary outcome |
| Ancillary analyses | 18 | Results of any other analyses performed, including subgroup analyses and adjusted analyses, distinguishing pre-specified from exploratory   - If subgroups analyses were undertaken, whether these were pre-specified - If subgroups analyses were undertaken, is it reported a test of interaction - If adjusted analyses were undertaken, whether these were pre-specified - If adjusted analyses were undertaken, is there a clear description of the of variables |  | □ Yes  □ Partial yes  □ No  □ N/A if none other analyses were performed |
| Harms | 19 | All important harms or unintended effects in each group |  | □ Yes  □ No |
| **Discussion** | | | | |
| Limitations | 20 | Trial limitations, including addressing sources of potential bias, imprecision, and if relevant, multiplicity of analyses |  | □ Yes  □ No |
| Generalizability | 21 | Generalizability (external validity, applicability) of the trial findings |  | □ Yes  □ No |
| Interpretation | 22 | Interpretation consistent with results, balancing benefits and harms, and considering other relevant evidence | - State the main findings of the article - the finding using intention-to-treat principles, with per-protocol interpretations - Discuss any relevant aspects of the active constituent of the intervention - Discuss the choice of comparator - Discuss any assessment of dietary adherence - Describe any potentially false discoveries due to any adjustments used in statistical analyses - Distinguish clearly between statistical and clinically relevant findings, with a detailed interpretation of how the findings affect clinical practice, dietary guidance, or public health | □ Yes  □ Partial yes  □ No |
| **Other information** | | | | |
| Registration | 23 | Registration number and name of trial registry |  | □ Yes  □ No |
| Protocol | 24 | Where the full trial protocol can be accessed, if available |  | □ Yes  □ No |
| Funding | 25 | Sources of funding and other support, role of funders |  | □ Yes  □ No |

20. Weaver C, Ahles S, Murphy KJ, Shyam S, Cade J, Plat J, et al. Perspective: Peer Evaluation of Recommendations for CONSORT Guidelines for Randomized Controlled Trials in Nutrition. Adv Nutr. 2024;15(1):100154.

Appendix 3 Included studies

1. Asaoka D, Xiao J, Takeda T, Yanagisawa N, Yamazaki T, Matsubara Y, et al. Effect of Probiotic Bifidobacterium breve in Improving Cognitive Function and Preventing Brain Atrophy in Older Patients with Suspected Mild Cognitive Impairment: Results of a 24-Week Randomized, Double-Blind, Placebo-Controlled Trial. J Alzheimers Dis. 2022;88(1):75-95.
2. Baltic S, Nedeljkovic D, Todorovic N, Ranisavljev M, Korovljev D, Cvejic J, et al. The impact of six-week dihydrogen-pyrroloquinoline quinone supplementation on mitochondrial biomarkers, brain metabolism, and cognition in elderly individuals with mild cognitive impairment: a randomized controlled trial. J Nutr Health Aging. 2024;28(8):100287.
3. Bo Y, Zhang X, Wang Y, You J, Cui H, Zhu Y, et al. The n-3 Polyunsaturated Fatty Acids Supplementation Improved the Cognitive Function in the Chinese Elderly with Mild Cognitive Impairment: A Double-Blind Randomized Controlled Trial. Nutrients. 2017;9(1).
4. Boespflug EL, Eliassen JC, Dudley JA, Shidler MD, Kalt W, Summer SS, et al. Enhanced neural activation with blueberry supplementation in mild cognitive impairment. Nutr Neurosci. 2018;21(4):297-305.
5. Chatzikostopoulos T, Gialaouzidis M, Koutoupa A, Tsolaki M. The Effects of Pomegranate Seed Oil on Mild Cognitive Impairment. J Alzheimers Dis. 2024;97(4):1961-70.
6. Choi WY, Lee WK, Kim TH, Ryu YK, Park A, Lee YJ, et al. The Effects of Spirulina maxima Extract on Memory Improvement in Those with Mild Cognitive Impairment: A Randomized, Double-Blind, Placebo-Controlled Clinical Trial. Nutrients. 2022;14(18).
7. Delfan M, Kordestani-Moghaddam P, Gholami M, Kazemi K, Mohammadi R. Evaluating the effects of Bacopa monnieri on cognitive performance and sleep quality of patients with mild cognitive impairment: A triple-blinded, randomized, placebo-controlled trial. Explore (NY). 2024;20(5):102990.
8. Desideri G, Kwik-Uribe C, Grassi D, Necozione S, Ghiadoni L, Mastroiacovo D, et al. Benefits in cognitive function, blood pressure, and insulin resistance through cocoa flavanol consumption in elderly subjects with mild cognitive impairment: the Cocoa, Cognition, and Aging (CoCoA) study. Hypertension. 2012;60(3):794-801.
9. Duan H, Xu N, Yang T, Wang M, Zhang C, Zhao J, et al. Effects of a food supplement containing phosphatidylserine on cognitive function in Chinese older adults with mild cognitive impairment: A randomized double-blind, placebo-controlled trial. J Affect Disord. 2025;369:35-42.
10. Duan H, Yang T, Li C, Xu N, Wang M, Zhang C, et al. Supplementation of medium-chain triglycerides combined with docosahexaenoic acid improves cognitive function in Chinese older adults with mild cognitive impairment: A randomized double-blind, placebo-controlled trial. J Affect Disord. 2025;378:263-70.
11. Fei Y, Wang R, Lu J, Peng S, Yang S, Wang Y, et al. Probiotic intervention benefits multiple neural behaviors in older adults with mild cognitive impairment. Geriatr Nurs. 2023;51:167-75.
12. Fortier M, Castellano CA, Croteau E, Langlois F, Bocti C, St-Pierre V, et al. A ketogenic drink improves brain energy and some measures of cognition in mild cognitive impairment. Alzheimers Dement. 2019;15(5):625-34.
13. Hu J, Jia J, Zhang Y, Miao R, Huo X, Ma F. Effects of vitamin D(3) supplementation on cognition and blood lipids: a 12-month randomised, double-blind, placebo-controlled trial. J Neurol Neurosurg Psychiatry. 2018;89(12):1341-7.
14. Hwang YH, Park S, Paik JW, Chae SW, Kim DH, Jeong DG, et al. Efficacy and Safety of Lactobacillus Plantarum C29-Fermented Soybean (DW2009) in Individuals with Mild Cognitive Impairment: A 12-Week, Multi-Center, Randomized, Double-Blind, Placebo-Controlled Clinical Trial. Nutrients. 2019;11(2).
15. Ito N, Saito H, Seki S, Ueda F, Asada T. Effects of Composite Supplement Containing Astaxanthin and Sesamin on Cognitive Functions in People with Mild Cognitive Impairment: A Randomized, Double-Blind, Placebo-Controlled Trial. J Alzheimers Dis. 2018;62(4):1767-75.
16. Jiang B, Yao G, Yao C, Zheng N. The effect of folate and VitB(12) in the treatment of MCI patients with hyperhomocysteinemia. J Clin Neurosci. 2020;81:65-9.
17. Jung SJ, Cho K, Jung ES, Son D, Byun JS, Kim SI, et al. Augmenting Cognitive Function in the Elderly with Mild Cognitive Impairment Using Probiotic Lacticaseibacillus rhamnosus CBT-LR5: A 12-Week Randomized, Double-Blind, Parallel-Group Non-Comparative Study. Nutrients. 2025;17(4).
18. Jung SJ, Jung ES, Ha KC, Baek HI, Park YK, Han SK, et al. Efficacy and Safety of Sesame Oil Cake Extract on Memory Function Improvement: A 12-Week, Randomized, Double-Blind, Placebo-Controlled Pilot Study. Nutrients. 2021;13(8).
19. Kaddoumi A, Denney TS, Jr., Deshpande G, Robinson JL, Beyers RJ, Redden DT, et al. Extra-Virgin Olive Oil Enhances the Blood-Brain Barrier Function in Mild Cognitive Impairment: A Randomized Controlled Trial. Nutrients. 2022;14(23).
20. Kang HJ, Lee EH, Choi SH, Moon SY, Jeong JH, Park YK. Effects of Oral Nutrition Supplementation with or Without Multi-Domain Intervention Program on Cognitive Function and Overall Health in Older Adults: A Randomized Controlled Trial. Nutrients. 2025;17(11).
21. Krikorian R, Nash TA, Shidler MD, Shukitt-Hale B, Joseph JA. Concord grape juice supplementation improves memory function in older adults with mild cognitive impairment. Br J Nutr. 2010;103(5):730-4.
22. Kwok T, Wu Y, Lee J, Lee R, Yung CY, Choi G, et al. A randomized placebo-controlled trial of using B vitamins to prevent cognitive decline in older mild cognitive impairment patients. Clin Nutr. 2020;39(8):2399-405.
23. Lau H, Shahar S, Mohamad M, Rajab NF, Yahya HM, Din NC, et al. The effects of six months Persicaria minor extract supplement among older adults with mild cognitive impairment: a double-blinded, randomized, and placebo-controlled trial. BMC Complement Med Ther. 2020;20(1):315.
24. Lee EH, Kim GH, Park HK, Kang HJ, Park YK, Lee HA, et al. Effects of the multidomain intervention with nutritional supplements on cognition and gut microbiome in early symptomatic Alzheimer's disease: a randomized controlled trial. Front Aging Neurosci. 2023;15:1266955.
25. Lee LK, Shahar S, Chin AV, Yusoff NA. Docosahexaenoic acid-concentrated fish oil supplementation in subjects with mild cognitive impairment (MCI): a 12-month randomised, double-blind, placebo-controlled trial. Psychopharmacology (Berl). 2013;225(3):605-12.
26. Li F, He R, Yue Z, Yi H, Lu L, Zhang L, et al. Effect of a 12-mo intervention with whey protein powder on cognitive function in older adults with mild cognitive impairment: a randomized controlled trial. Am J Clin Nutr. 2025;121(2):256-64.
27. Li M, Li W, Gao Y, Chen Y, Bai D, Weng J, et al. Effect of folic acid combined with docosahexaenoic acid intervention on mild cognitive impairment in elderly: a randomized double-blind, placebo-controlled trial. Eur J Nutr. 2021;60(4):1795-808.
28. Lin PY, Cheng C, Satyanarayanan SK, Chiu LT, Chien YC, Chuu CP, et al. Omega-3 fatty acids and blood-based biomarkers in Alzheimer's disease and mild cognitive impairment: A randomized placebo-controlled trial. Brain Behav Immun. 2022;99:289-98.
29. Liu W, Zheng D, Li X, Wang T, Wang L, Hao L, et al. Effects of Vitamin D3 Combined with Folic Acid on Domain and Specific Cognitive Function among Patients with Mild Cognitive Impairment: A Randomized Clinical Trial. J Prev Alzheimers Dis. 2024;11(6):1626-33.
30. Ma F, Li Q, Zhou X, Zhao J, Song A, Li W, et al. Effects of folic acid supplementation on cognitive function and Aβ-related biomarkers in mild cognitive impairment: a randomized controlled trial. Eur J Nutr. 2019;58(1):345-56.
31. Ma F, Zhou X, Li Q, Zhao J, Song A, An P, et al. Effects of Folic Acid and Vitamin B12, Alone and in Combination on Cognitive Function and Inflammatory Factors in the Elderly with Mild Cognitive Impairment: A Single-blind Experimental Design. Curr Alzheimer Res. 2019;16(7):622-32.
32. Masuoka N, Yoshimine C, Hori M, Tanaka M, Asada T, Abe K, et al. Effects of Anserine/Carnosine Supplementation on Mild Cognitive Impairment with APOE4. Nutrients. 2019;11(7).
33. May N, Fitzgerald Z, Broyd S, Rosario VD, Roodenrys S, Bliokas V, et al. Queen garnet plum juice supplementation does not provide additional cognitive benefits over a group-based memory program in older adults with mild cognitive impairment: A randomized clinical trial. Nutr Res. 2025;138:107-21.
34. McMaster M, Kim S, Clare L, Torres SJ, Cherbuin N, DʼEste C, et al. Lifestyle Risk Factors and Cognitive Outcomes from the Multidomain Dementia Risk Reduction Randomized Controlled Trial, Body Brain Life for Cognitive Decline (BBL-CD). J Am Geriatr Soc. 2020;68(11):2629-37.
35. Mengelberg A, Leathem J, Podd J, Hill S, Conlon C. The effects of docosahexaenoic acid supplementation on cognition and well-being in mild cognitive impairment: A 12-month randomised controlled trial. Int J Geriatr Psychiatry. 2022;37(5).
36. Montero-Odasso M, Zou G, Speechley M, Almeida QJ, Liu-Ambrose T, Middleton LE, et al. Effects of Exercise Alone or Combined With Cognitive Training and Vitamin D Supplementation to Improve Cognition in Adults With Mild Cognitive Impairment: A Randomized Clinical Trial. JAMA Netw Open. 2023;6(7):e2324465.
37. Mori K, Inatomi S, Ouchi K, Azumi Y, Tuchida T. Improving effects of the mushroom Yamabushitake (Hericium erinaceus) on mild cognitive impairment: a double-blind placebo-controlled clinical trial. Phytother Res. 2009;23(3):367-72.
38. Ochiai R, Saitou K, Suzukamo C, Osaki N, Asada T. Effect of Chlorogenic Acids on Cognitive Function in Mild Cognitive Impairment: A Randomized Controlled Crossover Trial. J Alzheimers Dis. 2019;72(4):1209-16.
39. Ornish D, Madison C, Kivipelto M, Kemp C, McCulloch CE, Galasko D, et al. Effects of intensive lifestyle changes on the progression of mild cognitive impairment or early dementia due to Alzheimer's disease: a randomized, controlled clinical trial. Alzheimers Res Ther. 2024;16(1):122.
40. Park KC, Jin H, Zheng R, Kim S, Lee SE, Kim BH, et al. Cognition enhancing effect of panax ginseng in Korean volunteers with mild cognitive impairment: a randomized, double-blind, placebo-controlled clinical trial. Transl Clin Pharmacol. 2019;27(3):92-7.
41. Park SK, Jung IC, Lee WK, Lee YS, Park HK, Go HJ, et al. A combination of green tea extract and l-theanine improves memory and attention in subjects with mild cognitive impairment: a double-blind placebo-controlled study. J Med Food. 2011;14(4):334-43.
42. Petersen RC, Thomas RG, Grundman M, Bennett D, Doody R, Ferris S, et al. Vitamin E and donepezil for the treatment of mild cognitive impairment. N Engl J Med. 2005;352(23):2379-88.
43. Power R, Nolan JM, Prado-Cabrero A, Coen R, Roche W, Power T, et al. Targeted Nutritional Intervention for Patients with Mild Cognitive Impairment: The Cognitive impAiRmEnt Study (CARES) Trial 1. J Pers Med. 2020;10(2).
44. Remington R, Lortie JJ, Hoffmann H, Page R, Morrell C, Shea TB. A Nutritional Formulation for Cognitive Performance in Mild Cognitive Impairment: A Placebo-Controlled Trial with an Open-Label Extension. J Alzheimers Dis. 2015;48(3):591-5.
45. Rita Cardoso B, Apolinário D, da Silva Bandeira V, Busse AL, Magaldi RM, Jacob-Filho W, et al. Effects of Brazil nut consumption on selenium status and cognitive performance in older adults with mild cognitive impairment: a randomized controlled pilot trial. Eur J Nutr. 2016;55(1):107-16.
46. Rondanelli M, Opizzi A, Faliva M, Mozzoni M, Antoniello N, Cazzola R, et al. Effects of a diet integration with an oily emulsion of DHA-phospholipids containing melatonin and tryptophan in elderly patients suffering from mild cognitive impairment. Nutr Neurosci. 2012;15(2):46-54.
47. Sakurai K, Toshimitsu T, Okada E, Anzai S, Shiraishi I, Inamura N, et al. Effects of Lactiplantibacillus plantarum OLL2712 on Memory Function in Older Adults with Declining Memory: A Randomized Placebo-Controlled Trial. Nutrients. 2022;14(20).
48. Sakurai T, Sugimoto T, Akatsu H, Doi T, Fujiwara Y, Hirakawa A, et al. Japan-Multimodal Intervention Trial for the Prevention of Dementia: A randomized controlled trial. Alzheimers Dement. 2024;20(6):3918-30.
49. Sinn N, Milte CM, Street SJ, Buckley JD, Coates AM, Petkov J, et al. Effects of n-3 fatty acids, EPA v. DHA, on depressive symptoms, quality of life, memory and executive function in older adults with mild cognitive impairment: a 6-month randomised controlled trial. Br J Nutr. 2012;107(11):1682-93.
50. Smith AD, Smith SM, de Jager CA, Whitbread P, Johnston C, Agacinski G, et al. Homocysteine-lowering by B vitamins slows the rate of accelerated brain atrophy in mild cognitive impairment: a randomized controlled trial. PLoS One. 2010;5(9):e12244.
51. Stavrinou PS, Andreou E, Aphamis G, Pantzaris M, Ioannou M, Patrikios IS, et al. The Effects of a 6-Month High Dose Omega-3 and Omega-6 Polyunsaturated Fatty Acids and Antioxidant Vitamins Supplementation on Cognitive Function and Functional Capacity in Older Adults with Mild Cognitive Impairment. Nutrients. 2020;12(2).
52. Suzuki T, Kojima N, Osuka Y, Tokui Y, Takasugi S, Kawashima A, et al. The Effects of Mold-Fermented Cheese on Brain-Derived Neurotrophic Factor in Community-Dwelling Older Japanese Women With Mild Cognitive Impairment: A Randomized, Controlled, Crossover Trial. J Am Med Dir Assoc. 2019;20(12):1509-14.e2.
53. Tadokoro K, Morihara R, Ohta Y, Hishikawa N, Kawano S, Sasaki R, et al. Clinical Benefits of Antioxidative Supplement Twendee X for Mild Cognitive Impairment: A Multicenter, Randomized, Double-Blind, and Placebo-Controlled Prospective Interventional Study. J Alzheimers Dis. 2019;71(3):1063-9.
54. Tsolaki M, Lazarou E, Kozori M, Petridou N, Tabakis I, Lazarou I, et al. A Randomized Clinical Trial of Greek High Phenolic Early Harvest Extra Virgin Olive Oil in Mild Cognitive Impairment: The MICOIL Pilot Study. J Alzheimers Dis. 2020;78(2):801-17.
55. Uchida K, Meno K, Korenaga T, Liu S, Suzuki H, Baba Y, et al. Effect of matcha green tea on cognitive functions and sleep quality in older adults with cognitive decline: A randomized controlled study over 12 months. PLoS One. 2024;19(8):e0309287.
56. Umeda K, Kobayashi K, Kanatome A, Sugimura Y, Ano Y, Suzuki H, et al. Effects of whey-derived lactopeptide β-lactolin on cognitive performance in mild cognitive impairment: a randomized, double-blind, placebo-controlled trial. Nutr Neurosci. 2024:1-11.
57. van Uffelen JG, Chinapaw MJ, van Mechelen W, Hopman-Rock M. Walking or vitamin B for cognition in older adults with mild cognitive impairment? A randomised controlled trial. Br J Sports Med. 2008;42(5):344-51.
58. Wang P, Yang T, Peng W, Wang M, Chen X, Yang Y, et al. Effects of a Multicomponent Intervention With Cognitive Training and Lifestyle Guidance for Older Adults at Risk of Dementia: A Randomized Controlled Trial. J Clin Psychiatry. 2024;85(2).
59. Xiao J, Katsumata N, Bernier F, Ohno K, Yamauchi Y, Odamaki T, et al. Probiotic Bifidobacterium breve in Improving Cognitive Functions of Older Adults with Suspected Mild Cognitive Impairment: A Randomized, Double-Blind, Placebo-Controlled Trial. J Alzheimers Dis. 2020;77(1):139-47.
60. Xu X, Shi D, Chen Y, Wang L, Jiang J, Xiao S. The Effects of Traditional Chinese Herbal Dietary Formula on the Ability of Daily Life and Physical Function in Elderly Patients with Mild Cognitive Impairment. Brain Sci. 2024;14(4).
61. Yakoot M, Salem A, Helmy S. Effect of Memo®, a natural formula combination, on Mini-Mental State Examination scores in patients with mild cognitive impairment. Clin Interv Aging. 2013;8:975-81.
62. Yang T, Wang H, Xiong Y, Chen C, Duan K, Jia J, et al. Vitamin D Supplementation Improves Cognitive Function Through Reducing Oxidative Stress Regulated by Telomere Length in Older Adults with Mild Cognitive Impairment: A 12-Month Randomized Controlled Trial. J Alzheimers Dis. 2020;78(4):1509-18.
63. You YX, Shahar S, Rajab NF, Haron H, Yahya HM, Mohamad M, et al. Effects of 12 Weeks Cosmos caudatus Supplement among Older Adults with Mild Cognitive Impairment: A Randomized, Double-Blind and Placebo-Controlled Trial. Nutrients. 2021;13(2).
64. Zhang YP, Miao R, Li Q, Wu T, Ma F. Effects of DHA Supplementation on Hippocampal Volume and Cognitive Function in Older Adults with Mild Cognitive Impairment: A 12-Month Randomized, Double-Blind, Placebo-Controlled Trial. J Alzheimers Dis. 2017;55(2):497-507.
65. Gavrilova SI, Preuss UW, Wong JW, Hoerr R, Kaschel R, Bachinskaya N. Efficacy and safety of Ginkgo biloba extract EGb 761 in mild cognitive impairment with neuropsychiatric symptoms: a randomized, placebo-controlled, double-blind, multi-center trial. Int J Geriatr Psychiatry. 2014;29(10):1087-95.
66. Grass-Kapanke B, Busmane A, Lasmanis A, Hoerr R, Kaschel R. Effects of Ginkgo Biloba Special Extract EGb 761 Very Mild Cognitive Impairment (vMCI). Neuroscience and Medicine. 2011;02(01):48-56.
67. Kato-Kataoka A, Sakai M, Ebina R, Nonaka C, Asano T, Miyamori T. Soybean-derived phosphatidylserine improves memory function of the elderly Japanese subjects with memory complaints. J Clin Biochem Nutr. 2010;47(3):246-55.
68. Köbe T, Witte AV, Schnelle A, Lesemann A, Fabian S, Tesky VA, et al. Combined omega-3 fatty acids, aerobic exercise and cognitive stimulation prevents decline in gray matter volume of the frontal, parietal and cingulate cortex in patients with mild cognitive impairment. Neuroimage. 2016;131:226-38.
69. Krikorian R, Eliassen JC, Boespflug EL, Nash TA, Shidler MD. Improved cognitive-cerebral function in older adults with chromium supplementation. Nutr Neurosci. 2010;13(3):116-22.
70. Krikorian R, Shidler MD, Dangelo K, Couch SC, Benoit SC, Clegg DJ. Dietary ketosis enhances memory in mild cognitive impairment. Neurobiol Aging. 2012;33(2):425.e19-27.
71. Naeini AM, Elmadfa I, Djazayery A, Barekatain M, Ghazvini MR, Djalali M, et al. The effect of antioxidant vitamins E and C on cognitive performance of the elderly with mild cognitive impairment in Isfahan, Iran: a double-blind, randomized, placebo-controlled trial. Eur J Nutr. 2014;53(5):1255-62.
72. Xu L, Yu H, Sun H, Hu B, Geng Y. Dietary Melatonin Therapy Alleviates the Lamina Cribrosa Damages in Patients with Mild Cognitive Impairments: A Double-Blinded, Randomized Controlled Study. Med Sci Monit. 2020;26:e923232.
73. Horie NC, Serrao VT, Simon SS, Gascon MR, Dos Santos AX, Zambone MA, et al. Cognitive Effects of Intentional Weight Loss in Elderly Obese Individuals With Mild Cognitive Impairment. J Clin Endocrinol Metab. 2016;101(3):1104-12.
74. Johari SiM, Shahar S, Ng TP, Rajikan R. A Preliminary Randomized Controlled Trial of Multifaceted Educational Intervention for Mild Cognitive Impairment Among Elderly Malays in Kuala Lumpur. International Journal of Gerontology. 2014;8(2):74-80.
75. Kobayashi Y, Kuhara T, Oki M, Xiao JZ. Effects of Bifidobacterium breve A1 on the cognitive function of older adults with memory complaints: a randomised, double-blind, placebo-controlled trial. Benef Microbes. 2019;10(5):511-20.
